# Co-FeS_2_/CoS_2_ Heterostructured Nanomaterials for pH Sensing

**DOI:** 10.3390/s20195571

**Published:** 2020-09-29

**Authors:** Yuan Gao, Zehui Peng, Ka Wang, Shancheng Yan, Zixia Lin, Xin Xu, Yi Shi

**Affiliations:** 1 School of Geography and Biological Information, Nanjing University of Posts and Telecommunications, Nanjing 210023, China; 1218063835@njupt.edu.cn (Y.G.); 1019172222@njupt.edu.cn (Z.P.); 1217063729@njupt.edu.cn (K.W.); xuxin@njupt.edu.cn (X.X.); 2 Testing Center, Yangzhou University, Yangzhou 225009, China; zzxlin@yzu.edu.cn; 3 School of Electronic Science and Engineering, Nanjing University, Nanjing 210093, China; yshi@nju.edu.cn

**Keywords:** alkali sensor, hydrogen evolution reaction, heterostructure, electrocatalyst

## Abstract

Biosensors are widely used in production and life, and can be used in medicine, industrial production, and scientific research. Among them, the detection of pH has always received extensive attention. In this study, we demonstrate the use of a one-step hydrothermal method to prepare Co-FeS_2_/CoS_2_ nanomaterials as pH sensor (pH vs. overpotential) for the first time. The proposed pH sensor exhibits outstanding performance in KOH solutions via electrochemical methods with good stability. Overall, the results of this study not only add to the non-noble transition metal electrocatalysis research, but also identify important sensing characteristics for electrocatalysts.

## 1. Introduction

From the perspective of the environment protection and human health, some sensors used to monitor temperature, moderation, biological substances, and heavy metals have a vital development trend. Non-enzymatic hydrogen peroxide (H_2_O_2_) sensor was fabricated, which is based on few-layer black phosphorus (BP) prepared by supercritical carbon dioxide, to utilize BP degradation under ambient conditions [1]. BP can be used to detect biological molecules such as immunoglobulin G (IgG), DNA, and carcinoembryonic antigen (CEA) [2]. In a narrow sense, many biological and chemical reactions depend on the pH value. pH sensors are widely used in production and life processes to ensure human health, water quality, food quality, and monitor chemical or biological reactions [3,4]. Accurate pH determination has always been an important part of life and production. Semiconductor polymer dots can also be used as sensitive and broad-range photoelectrochemical pH sensors. Changes in pH will cause conformational changes and further diffusion of carries. The redox characteristics of polymer dots will also change. These will cause the photocurrent generated by the electrode to change [5]. Metal compounds are often a good choice for water quality testing and research when using electrochemical workstation [6,7]. A pH sensor was obtained by using screen printing of TiO_2_ thick film on alumina substrate. Additionally, it can be observed easily that the impendence of the thick film is distinctly dependent on pH. However, the other type of the pH sensor such as low cost, advanced materials still need to be developed or improved. In renewable energy fields, the electrolysis of water is an important way to produce hydrogen [8,9,10,11]. Now, non-noble transition metal has been studied in the electrolysis of water [12,13,14]. However, the research on using catalyst materials as pH detection sensors, which remains rare.

In this study, we adopt a simple strategy to synthesize Co-FeS_2_/CoS_2_ nanomaterials with good selectivity which is easy to prepare, low in cost, simple in testing, good in selectivity and reliable in results [15,16]. Using these Co-FeS_2_/CoS_2_ nanomaterials, a pH sensor was constructed to utilize the electrocatalytic overpotential at different pH values. The overpotential of the hydrogen evolution reaction of Co-FeS_2_/CoS_2_ nanoflowers in KOH solutions of different concentrations was tested and a functional relationship between the pH value of the solution and hydrogen evolution was constructed. Our proposed pH sensor exhibits outstanding performance in alkaline solutions via electrochemical methods. The results of this study not only add to the non-noble transition metal electrocatalysts research range but also identify important sensing characteristics for electrocatalysts. In the following research, there is hope that Co-FeS_2_/CoS_2_ will be used for heavy metal ion detection.

## 2. Materials and Methods

### 2.1. Materials and Chemicals

FeSO_4_·7H_2_O used in the experiment was purchased from Shanghai Titan Technology Co., Ltd. (Shanghai, China) Purchased sublimation sulfur (S), SC(NH_2_)_2_, Co(NO_3_)_2_·6H_2_O, KOH, C_2_H_5_OH from Nanjing Chemical Reagent Co., Ltd. (Nanjing, China). Ultrapure water is obtained through Millipore pure water filters (Millipore Q, Billerica, MA, USA). WOS1009 carbon cloth (CC) was provided by CeTech Co., Ltd. (Taichung County, Taiwan).

### 2.2. Preparation of Co-FeS_2_/CoS_2_ Heterostructure Nanomaterials

In this experiment, the carbon cloth (2 cm × 2 cm) was ultrasonically cleaned with deionized water and absolute ethanol for 15 min, and then the carbon cloth was blow dried with a hot air blower. Subsequently, SC(NH_2_)_2_ (1.8 mM), FeSO_4_·7H_2_O (1.2 mM), and Co(NO_3_)_2_·6H_2_O (0.156 mM) and 25 mL of deionized water were added to the 50 mL polytetrafluoroethylene reactor. The reaction kettle was placed on a magnetic stirrer and stirred at a higher speed for 15 min to form uniform and transparent solution. Then, during the stirring process, 0.96 mmol of sulfur powder was slowly poured into the above reaction kettle, and the stirring was continued for 10 min after reducing the speed of the stirrer. After the stirring was stopped, the sulfur powder would form a thin film on the solution. The cleaned carbon cloth was put horizontally in the reaction kettle solution; the reaction kettle was tightened and put in a 180 °C blast-drying oven for 8 h of reaction.

### 2.3. Eelectrochemical Studies

The CHI760E electrochemical analyzer (CH instrument, Shanghai Chenhua Company, Shanghai, China) was used for electrochemical measurement. The sample was used as the working electrode, the calomel electrode was used as the reference electrode, and the graphite rod was used as the counter electrode. KOH solution was used as electrolyte solution and oxygen contained in the solution was removed by bubbling nitrogen before testing. Linear scan voltammetry (LSV) has a scan rate of 2 mV S^−1^. The corresponding Tafel slope is calculated according to the logarithmic relationship between the overpotential and current density in the LSV curve.

## 3. Results and Discussion

### 3.1. XRD Result

**Figure 1:**
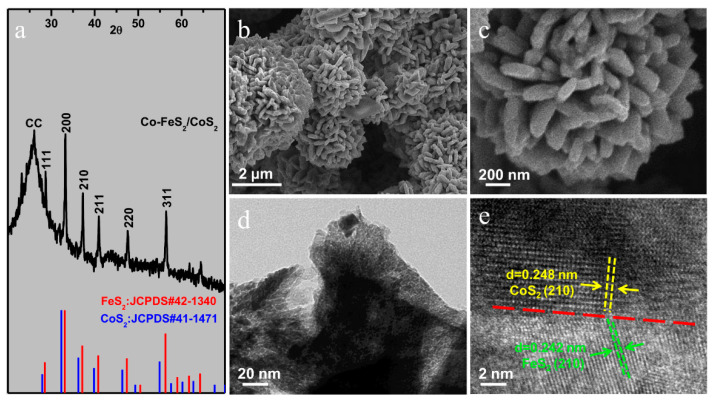
(**a**) The X-ray diffraction (XRD) patterns for Co-FeS_2_/CoS_2_ heterostructure; (**b**,**c**) SEM images of Co-FeS_2_/CoS_2_ heterostructure; (**d**) TEM image of Co-FeS_2_/CoS_2_ heterostructure; (**e**) HRTEM image of Co-FeS_2_/CoS_2_ heterostructure.

Figure 1a shows the X-ray diffraction (XRD) pattern of the Co-FeS_2_/CoS_2_ heterostructure. It can be seen from the XRD pattern that the Co-FeS_2_/CoS_2_ exhibits good crystallinity. The broad peak at 26.5° belongs to the carbon cloth [17]. The six peaks at 28.4°, 33.2°, 37.3°, 40.9°, 47.6°, and 56.4° are due to FeS_2_ (JCPDS#42-1340) and CoS_2_ (JCPDS#41-1471) [18,19], corresponding to the (111), (200), (210), (211), (220), and (311) planes of FeS_2_, respectively.

### 3.2. SEM Results

As shown in the SEM image of Figure 1b, the micro-morphology of Co-FeS_2_/CoS_2_ is flower-like, with a diameter of about 5 micrometers. This clearly shows the nano-flower structure of the Co-FeS_2_/CoS_2_ heterostructure. The nano-petals on the flowers are interwoven and connected to form a 3D micro-flower structure [20]. This structure not only greatly increases the specific surface area, but also enhances the activity. More active sites can make the hydrogen produced by the decomposition of water during the reaction easier to desorb and adsorb, which can improve the performance of electrocatalyst. Figure 1c is a high-resolution SEM image of Co-FeS_2_/CoS_2_ heterostructure. The presence of CoS_2_ on the nano-petals makes the surface uneven, which also further increases its specific surface area [21,22,23].

### 3.3. TEM Results

TEM image of Co-FeS_2_/CoS_2_ heterostructure nano-petals is shown in Figure 1d. CoS_2_ was observed in the nano-petals, further increasing the surface area of the sample and helps to adjust the kinetic barrier in the hydrogen evolution reaction. Figure 1e shows a high-resolution TEM (HRTEM) image of the Co-FeS_2_/CoS_2_ heterostructure which demonstrates lattice fringes with spacing of 0.248 and 0.242 nm, corresponding to the (210) facet of the cubic CoS_2_ and the (210) facet of the cubic FeS_2_, respectively. The (210) interplanar spacing of FeS_2_ is 0.24 nm [24], and the (210) interplanar spacing of CoS_2_ is 0.25 nm [17]. The same crystal configuration and the similar interplanar spacing of FeS_2_ and CoS_2_ provide favorable conditions for the formation of heterostructures of FeS_2_ and CoS_2_. This strongly supports the Co-FeS_2_/CoS_2_ heterostructure to exhibit superior electrocatalytic hydrogen absorption performance. In other words, the Co-FeS_2_/CoS_2_ heterostructure is more responsive to the solution environment change such as pH value fluctuation.

### 3.4. XPS Results

**Figure 2:**
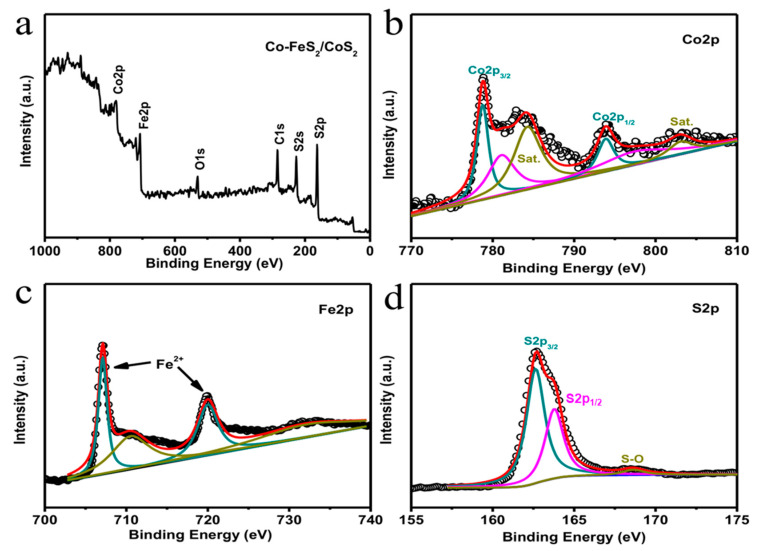
XPS spectra of Co-FeS_2_/CoS_2_ heterostructure: (**a**) survey spectrum, and high-resolution, (**b**) Co 2p, (**c**) Fe 2p, (**d**) S 2p spectrum.

X-ray photoelectron spectroscopy (XPS) was used to study the chemical composition and elemental oxidation states of the Co-FeS_2_/CoS_2_ heterostructure. The XPS survey spectrum in Figure 2a shows that the Co-FeS_2_/CoS_2_ heterostructure is mainly composed of Co, Fe, and S. Figure 2b shows the high-resolution XPS spectrum of Co 2p. From the peak splitting results, Co 2p is split into three spin-orbit doublets. The two peaks with binding energies of 778.7 and 794.1 eV belong to cobalt in Co-FeS_2_. The fitted peaks at 780.9 eV and 797.3 eV can be assigned to cobalt in single-phase CoS_2_ [25]. The two satellite peaks (identified as “Sat”) with binding energies at 784.8 and 803.3 eV are attributed to oxidized Co, produced by the oxidation of the Co-FeS_2_/CoS_2_ surface [26]. Figure 2c shows the high-resolution XPS spectrum of Fe 2p. The peaks at 707.8 and 720.5 eV belong to Fe 2p_3/2_ and Fe 2p_1/2_, respectively [27]. While the peaks at 711.2 and 732.5 eV belong to oxides of FeS_2_ on the surface of the Co-FeS_2_/CoS_2_ heterostructure [28]. The high-resolution S 2p XPS spectrum, shown in Figure 2d, has a peak at 162.6 eV, which belongs to S_2_^2−^ in FeS_2_ [29]. The peak at 163.8 eV belongs to the S in the Co-FeS_2_ structure, while the peak at 168 eV is attributed to the oxide species of S [30].

### 3.5. Electrocatalytic Performace

**Figure 3:**
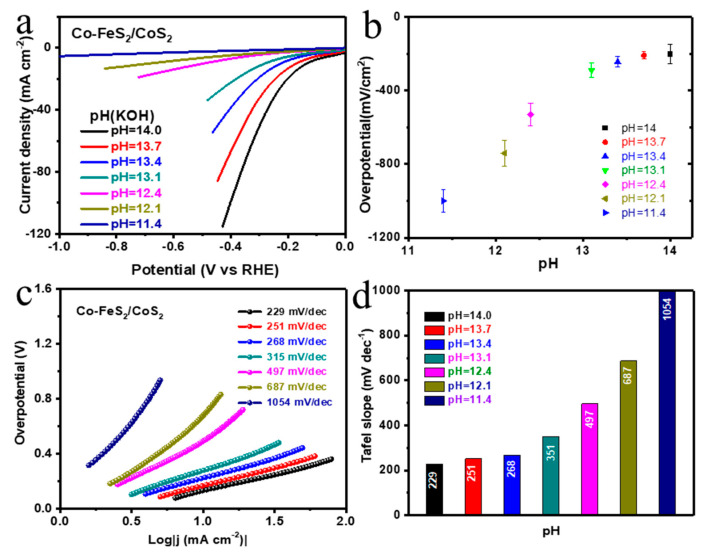
(**a**) LSV polarization curve of Co-FeS_2_/CoS_2_ heterostructure in KOH solutions of different pH; (**b**) Scatter plot corresponding to overpotential at current density 10 mV cm^−2^ in (**a**); (**c**) Tafel diagram of Co-FeS_2_/CoS_2_ heterostructure in KOH solutions of different pH; (**d**) Histogram corresponding to the slope of Tafel in (**c**).

The electrocatalytic performance of Co-FeS_2_/CoS_2_ nanoflowers was tested in KOH solutions of different pH. As shown in Figure 3a, when the pH of the solution is 14, 13.7, 13.4, 13.1, 12.4, 12.1, and 11.4, the overpotentials required to reach a current density of 10 mA cm^−2^ are 132, 168, 223, 279, 492, 678, and > 1000 mV, respectively. By comparing the overpotential at different pH, it can be found that the overpotential changes significantly with the change of pH which has a higher sensitivity [31,32]. As shown in Figure 3b, to further analyze the relationship between current density and pH, the required overpotential at a current density of 10 mA cm^−2^ and different pH values were plotted. The equation obtained by linear fitting is y = 0.322x − 4.6 (R^2^ = 0.944).

The results manifested the high sensitivity of the developed electrocatalytic sensor. When testing the pH of an unknown solution, the overpotential required for Co-FeS_2_/CoS_2_ nano-flowers in this solution, at a current density of 10 mA cm^−2^, can be measured first, and the pH of the unknown solution can be obtained by substituting the overpotential into the equation. As a pH sensor, adding other cations to the solution (pH = 14) will not interfere with the results, and there is almost no change in overpotential. The Tafel slope is an important indicator for evaluating the reaction rate during the HER process. It reveals the additional voltage required when the current density increases by a factor of 10. As shown in Figure 3c, the required Tafel slopes of the Co-FeS_2_/CoS_2_ nano-flowers are 229, 251, 268, 315, 497, 687 and 1054 mV dec^−1^, when the solution pH is 14, 13.7, 13.4, 13.1, 12.4, 12.1 and 11.4, respectively. As shown in Figure 3d, a histogram was created to reveal the relationship between the Tafel slope and pH more intuitively. Establishing a reliable linear relationship between the electrocatalytic values of Co-FeS_2_/CoS_2_ nanoflowers and the pH of the solution can be used to obtain the pH of an unknown solution.

**Figure 4:**
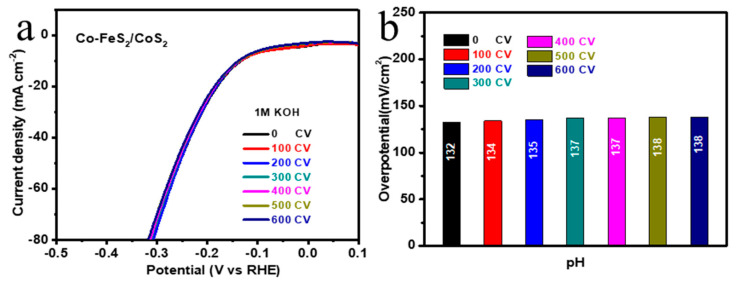
(**a**) LSV curve of Co-FeS_2_/CoS_2_ heterostructure after 0 to 600 CV cycles; (**b**) Histogram corresponding to the overpotential of the LSV curve of the Co-FeS_2_/CoS_2_ heterostructure at 10 mV cm^−2^ after 0 to 600 CV cycles in (**a**).

**Figure A1:**
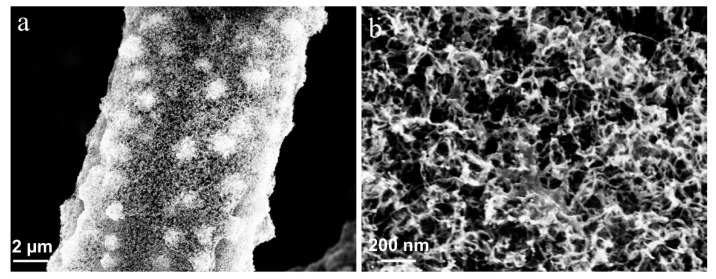
(**a**,**b**) SEM images of Co-FeS_2_/CoS_2_ nanowires.

**Figure A2:**
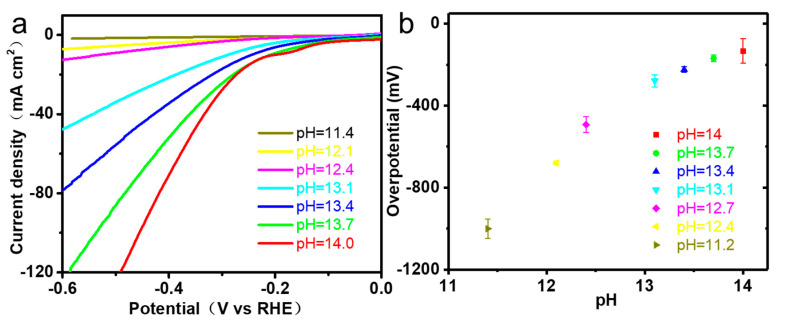
(**a**) LSV polarization curve of Co-FeS_2_/CoS_2_ nanowires in KOH solutions of different pH; (**b**) Scatter plot corresponding to overpotential at current density 10 mV cm^−2^ in (**a**).

Due to adsorption/diffusion of H^+^/OH^−^ ions the surface of nanoflowers becomes charged and it creates an electrical double layer structure by site binding theory. With changes in the pH of a solution the contribution of both H^+^ and OH^−^ ions also varies, which can affect the efficiency of HER [13]. Also, Figure A1 shows that Co-FeS_2_/CoS_2_ nanowires synthesized in previous work were used as a pH sensor, and a similar linear relationship was obtained [20]. Figure A2 shows that by plotting the overpotential required for the Co-FeS_2_/CoS_2_ nanowires to reach a current density of 10 mA cm^−2^ versus pH, the linear equation y = 0.333x − 4.7 was obtained (R^2^ = 0.953). Both Co-FeS_2_/CoS_2_ nanoflowers and Co-FeS_2_/CoS_2_ nanowires have the potential to be used as pH sensors. In order to ensure the applicability of the material, we conducted a stability test of 0–600 cycles on the samples, and performed an electrocatalytic activity test every 100 cycles. The resulting LSV curve is shown in Figure 4a. Figure 4b is the overpotential corresponding to the curve in Figure 4a after each stability test. It can be seen that after the stability test, the performance only decreased 6 mV, indicating the good reversibility, which is the better reversibility than the previous work [12,13,31].

## 4. Conclusions

In short, we prepared Co-FeS_2_/CoS_2_ heterostructure nano-flowers by a hydrothermal method and the hydrogen evolution performance of Co-FeS_2_/CoS_2_ nano-flowers were tested in KOH solutions of differing concentrations. By plotting the pH values of different solutions and the overpotential required for hydrogen evolution, it was found that all parameters are distinctly pH dependent. Due to the excellent stability in alkaline solution, the stable pH sensor exhibits outstanding performance such as selectivity and reproducibility. The relationship between them provides a new strategy for testing and analyzing the pH of unknown solutions.
